# Synthesis of a Novel Biodegradable Polyurethane with Phosphatidylcholines

**DOI:** 10.3390/ijms11041870

**Published:** 2010-04-26

**Authors:** Jun Cao, Niancao Chen, Yuanwei Chen, Xianglin Luo

**Affiliations:** 1 College of Polymer Science & Engineering, Sichuan University, Chengdu 610065, China; E-Mails: caojun198610301981@126.com (J.C); chenncscu@gmail.com (N.C.); chenyuanweizm@163.com (Y. C.); 2 State Key Lab of Polymer Materials and Engineering, Sichuan University, Chengdu 610065, China

**Keywords:** polyurethanes, phosphatidylcholine, biodegradable, blood compatibility, polymer, surface

## Abstract

A novel polyurethane was successfully synthesized by chain-extension of biodegradable poly (l-lactide) functionalized phosphatidylcholine (PC) with hexamethylene diisocyanate (HDI) as chain extender (PUR-PC). The molecular weights, glass transition temperature (Tg) increased significantly after the chain-extension. The hydrophilicity of PUR-PC was better than the one without PC, according to a water absorption test. Moreover, the number of adhesive platelets and anamorphic platelets on PUR-PC film were both less than those on PUR film. These preliminary results suggest that this novel polyurethane might be a better scaffold than traditional biodegradable polyurethanes for tissue engineering due to its better blood compatibility. Besides, this study also provides a new method to prepare PC-modified biodegradable polyurethanes.

## 1. Introduction

Biomaterials for tissue regeneration need to be biocompatible as well as biodegradable *in vivo*. Polyurethanes (PURs) are widely used in biomedical fields due to their excellent mechanical properties [1]. In order to further improve their blood compatibility, lots of modification methods have been developed. Among them, the imitation of cell membrane by introducing PC into PURs is a very effective way [2–5]. For example, aryl azides consisting of a photoactivatable 4-azidobenzoyl group and a PC end group have been synthesized and then the PC-containing aryl azides were coupled to poly (etherurethane) surfaces. Many studies purport to assess blood compatibility by counting adherent platelets and changing the morphology of the platelets on the surfaces of materials. What is more, as soon as the blood contacts a material, the protein starts to adsorb onto the materials surface and change their conformation. Thus, the platelets interact with these and eventually starting adhering and spreading onto the protein layer. The platelets in the state are activated to release blood coagulation factors [6]. So, the blood compatibility of the modified PURs was evaluated with a thrombin generation assay and a platelet adhesion study. The maximum thrombin concentration was lower than that of the original PURs. Meanwhile, the clotting time of the blood was also extended and platelet adhesion was effectively reduced on the modified PURs [4,5]. However, all the membrane-like PURs reported so far are seldom likely to degrade, which becomes the main limitation for their further application in tissue engineering. Although biodegradable PURs without PC have been intensively investigated in recent years, such as poly (lactide) (PLA), poly(glycolic acid) (PGA), poly(caprolactone) (PCL) and their copolymers *etc.* [7–11], they are not so blood compatible compared to PC-containing PURs.

In this study, PUR-PC, a novel polyurethane with PC groups in the biodegradable soft segments (PC-S) was synthesized. Meanwhile, polyurethane (PUR), as a contrast, was synthesized in the same way using PLLA as soft segment (S). The molecular structure and molecular weight were analyzed by proton NMR (^1^H-NMR), Fourier transform infrared spectroscopy (FT-IR) and Size exclusion chromatograph (SEC), respectively. Differential scanning calorimetry (DSC) was used to monitor the thermal properties. The hydrophilicity and *in vitro* blood compatibility were evaluated by water absorption test and platelet adhesion test respectively.

## 2. Experiment

**Figure 1.**
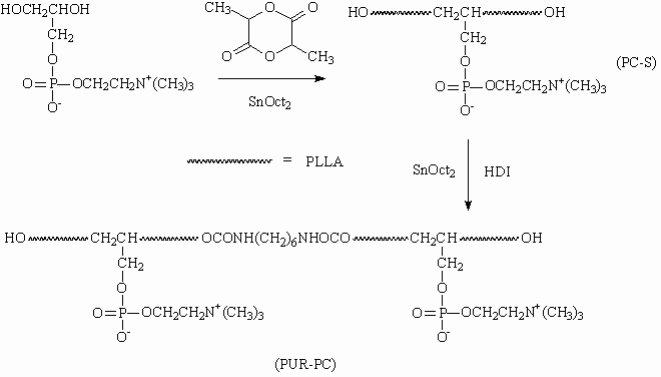
Synthetic route to PUR-PC.

The synthesis of PC-S was carried out under vacuum at 120 °C for 36 h by using glycerophosphatidylcholine as initiator (Figure1) [12]. Then PC-S was dried for two hours under vacuum before introducing it into a three-neck flask with toluene, a magnetic stir bar and stannous octoate (catalyst). The prescribed amount of hexamethylene diisocyanate (HDI) in toluene was added into the flask dropwise (molar ratio of HDI/prepolymer = 3). The reaction progressed under a N_2_ atmosphere at 120 °C for 7 h. The products were precipitated in ethanol and dried under vacuum to a constant weight. Synthesis of PUR without PC was carried out in a similar way. Figure 1 illustrates the synthesis of PC-S and PUR-PC.

Polymers were characterized by ^1^H-NMR (Bruker AV II-400 MHz), FT-IR (Nicolet 560). SEC was performed on equipment composed of a Waters 510 HPLC pump, a Waters 410 differential refractometer and a PLgel 5 mm mixed-C 60 cm column, the mobile phase being tetrahydrofuran (THF) and the flow rate 1 mL/min. The number-average (Mn) and weight-average (Mw) molecular weights were expressed with respect to polystyrene standards obtained from Polysciences. DSC (Netzsch DSC204) was used to characterize the glass transition temperature (Tg).

Water absorption was used to evaluate the hydrophilicity. It is defined as the weight percentage of water in wet polymer films. The films were prepared by casting 1 wt% polymer solution (in chloroform) onto polyethyleneglycol terephthalate (PET) substrates (1 cm × 1 cm). After vacuum drying for 2 days, the films were weighed (*W*_0_) and placed in distilled water. They were recovered at different time intervals, carefully wiped with filter paper and weighed (*W*_1_) again. The water absorption was calculated with the following equation:

$$\text{Water}\;\text{uptake}\;(\%)\;=\;(W_{1}-W_{0})/W_{0}\times 100\%$$

The blood platelet adhesion evaluation was carried out according to the method reported by reference [2]. Films were sputter-coated with gold in vacuum and observed by using a scanning electron microscope (SEM, JSM- 5900 LV, Jeol Ltd., Japan).

## 3. Experimental Results and Discussion

### 3.1. The Structure and Characterization of the POLYMERs

**Figure 2.**
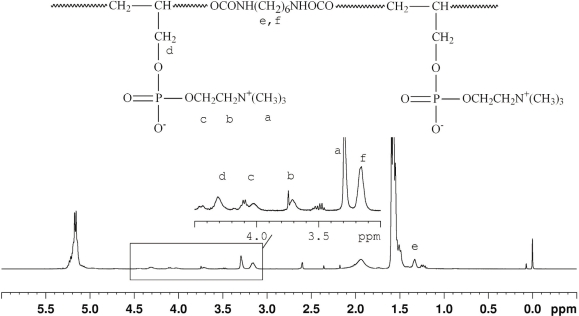
^1^H-NMR spectrum of PUR-PC.

To obtain PC-modified polyurethane, both surface modification and bulk modification methods are developed by researchers. In the bulk modification, PC is normally introduced by a graft reaction after chain extension. In addition, PC could also be introduced during the chain-extending reaction. In this situation, PC-containing chemicals served as chain-extenders [3]. In this study, a biodegradable polyurethane with PC (PUR-PC) was synthesized according to the synthetic route demonstrated in Figure 1. The synthesis strategy is similar to that of the normal polyurethane, which includes the synthesis of soft segments initially and then the chain-extension. PLLA with PC (PLLA/PC) took the role of the soft segment, the part of polylactide (PLA) is widely recognized to be biodegradable in the physiological environment. PLLA/PC was prepared by ring-opening polymerization of lactide with glycerophosphorylcholine as initiator (see Figure 1). The molecular structures were confirmed by the results of FT-IR, ^13^C-NMR and ^1^H-NMR analysis which were consistent with those reported before [12] (data not shown). Chain extension was then carried out after the confirmation. HDI served as the chain-extender (see Figure1). FT-IR and ^1^H-NMR were also employed to analyze the molecular structure of the obtained polyurethane. Figure 2 shows the ^1^H-NMR spectrum of PUR-PC. Signals at 1.55 and 5.2 were ascribed to the -CH_3_ and -CH_2_- groups in the PLLA blocks in the soft segments, respectively. The typical peaks of PC, including -N^+^(CH_3_)_3_, -OPOCH_2_- and -N^+^CH_2_- were found at 3.25, 3.43 and 4.3, respectively [12,13]. Signals at 1.3 and 3.15 were assigned to the -CH_2_- units of HDI in the hard segments [14]. In FT-IR spectra, the characteristic absorption peak of -N^+^(CH_3_)_3_ in the soft segment was observed at 970 cm^−1^ [12,13]. The peaks at 1,528 cm^−1^ and 3,405 cm^−1^ were attributed to -NH-and NH-CO, respectively, which were absent in the spectrum of soft segments.

The molecular weights (M_W_) of the synthesized polymers were examined by SEC. As shown in Table 1, the number average M (Mn) of the soft segment PC-S was about 2,500, it increased to more than 5,000 after the chain-extending reaction. This result once again confirmed the successful synthesis of PUR-PC polymer. DSC was employed to test the thermal properties of the polymers. The glass transition temperature (Tg) values of the soft segment of the PUR-PC was only about 28 °C. It increased to 53.3 °C after the chain extension (the chain-extender (see Table 1). These might be explained by the improved molecular weight and by the strong interaction between molecular chains resulting from the polarity of the O=C=N groups in the polyurethane.

### 3.2. Water Absorption and Blood Platelet Adhesion Test

**Figure 3.**
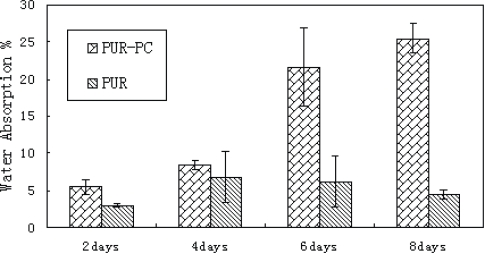
Water absorption of the synthesized copolymers.

Water absorption was used to evaluate the hydrophilicity of the polymers. As shown in Figure 3, the water absorption value of PUR-PC was about 6% 2 days after being in water. It’s evidently higher than that of the PUR without PC. Moreover, this difference increased over time durin the period of 8 days. At the end of the test period, the water absorption value of PUR-PC was about five times of the PUR water absorption value. This suggested that biodegradable PUR-PC was more hydrophilic than PUR. Normally, the introduction of PC would improve the hydrophilicity of polymers. This was ascribed to strong affinity between PC and water molecules. Various published papers have proved that the PC groups migrate to the surfaces of polymers when contacted with water and consequently combined with water molecules [12,13,15,16].

**Figure 4.**
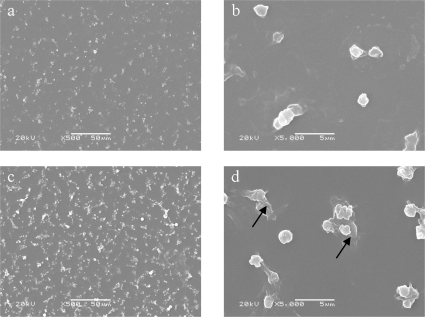
SEM pictures of platelets adhered on polymers surface. Black arrows: Anamorphic platelets: a, b – PUR-PC; c, d – PUR.

Figure 4 shows the adherent platelets (white dots) on the polymer surface after contacting with rabbit platelet rich plasma (PRP) for 60 min. A large number of platelets were observed to have adhered on PUR. In contrast; much less platelets could be seen on PUR-PC surface. Moreover, cell morphologies were found to be different on both of the polyurethanes films. Some platelets on PUR protruded flake pseudopodias, which made them larger than the static platelets (black arrows in Figure 4). In contrast, almost all the platelets on PUR-PC remained in their inactive round shape. Therefore, it could be deduced that PUR-PC could diminish adhesion and activation of platelets on PUR surface. This might be attributed to the affinity between the water molecules and the polar PC in PUR-PC. And this led to the formation of a layer of water on the surfaces of polymers in aqueous environment. The water layer suppressed the non-specific protein adsorption and subsequent platelet adhesion, as reported in references [2,4,5].

## 4. Conclusions

A novel polyurethane with PC in its biodegradable soft chains was successfully synthesized. The molecular weight and glass transition temperature (Tg) increased significantly after the chain-extension. The hydrophilicity of PUR-PC was better than that of PUR. Moreover, the number of adhesive platelets and anamorphic platelets on PUR-PC were both less than those on PUR. These preliminary results suggested that the novel polyurethane might be a better scaffold than traditional biodegradable PUR for tissue engineering due to its better blood compatibility. This study also provides a new method to prepare PC-modified biodegradable PUR.

## Figures and Tables

**Table 1.** Characterization of the polymers.

**Polymers**	**Mn a)**	**Mw b)**	**PDI b)**	**Tg (°C)**
PC-S c)	2490	3680	1.48	27.7
PUR-PC	5460	8270	1.52	53.3
PC-S’	3153	3996	1.267	38.6
PUR-PC’	8234	14532	1.765	55.2
PUR	24380	57680	2.37	44.7

a) Calculated by SEC results;

b) The polydispersity index, PDI = Mw/Mn;

c) The soft segment of PUR-PC, LLA/PC molar ratio in feed was 33/1.

## References

[1] Guelcher SA Biodegradable polyurethanes: synthesis and applications in regenerative medicine Tissue Eng.: B 2008 14 3 17 10.1089/teb.2007.0133 18454631

[2] Korematsua A Takemotob Y Nakaya T Inouea H Synthesis, characterization and platelet adhesion of segmented polyurethanes grafted phospholipid analogous vinyl monomer on surface Biomaterials 2001 23 263 271 10.1016/s0142-9612(01)00104-1 11762845

[3] Baumgartner JN Yang CZ Cooper SL Physical property analysis and bacterial adhesion on a series of phosphonated polyurethanes Biomaterials 1997 18 831 837 9184746 10.1016/s0142-9612(96)00197-4

[4] van der Heiden AP Goeggels D Pijpers AP Koole LH A photochemical method for the surface modification of poly (etherurethanes) with phosphorylcholine-containing compounds to improve hemocompatibility J. Biomed. Mater. Res 1997 37 282 290 9358323 10.1002/(sici)1097-4636(199711)37:2<282::aid-jbm19>3.0.co;2-g

[5] van der Heiden AP Willems GM Lindhout T Pijpers AP Koole LH Adsorption of proteins onto poly(ether urethane) with a phosphorylcholine moiety and influence of preadsorbed phospholipid J Biomed Mater Res 1998 40 195 203 9549614 10.1002/(sici)1097-4636(199805)40:2<195::aid-jbm4>3.0.co;2-g

[6] Ratner BD The catastrophe revisted: Blood compatibility in the 21st Century Biomaterials 2007 28 5144 5147 17689608 10.1016/j.biomaterials.2007.07.035 PMC2048822

[7] Guan JJ Fujimoto KL Sacks MS Wagner WR Preparation and characterization of highly porous, biodegradable Polyurethane scaffolds for soft tissue applications Biomaterials 2005 26 3961 3971 15626443 10.1016/j.biomaterials.2004.10.018 PMC2857583

[8] Alperina C Zandstrab PW Woodhouse KA Polyurethane films seeded with embryonic stem cell-derived cardio myocytes for useincardiac tissue engineering applications Biomaterials 2005 26 7377 7386 16023195 10.1016/j.biomaterials.2005.05.064

[9] Gorna K Gogolewski S Biodegradable porous polyurethane scaffolds for tissue repair and regeneration J. Biomed. Mater. Res 2006 79A 128 138 10.1002/jbm.a.30708 16779769

[10] Wang WS Ping P Chen XS Jing XB Biodegradable polyurethane based on random copolymer of L-lactide and ɛ-caprolactone and its shape-memory property J. Appl. Polym. Sci 2007 104 4182 4187

[11] Da Silva GR Da Silva Cunha AS Jr Ayres E Orefice RL Effect of the macromolecular architecture of biodegradable polyurethanes on the controlled delivery of ocular drugs J. Mater. Sci.: Mater. Med 2009 20 481 487 18853235 10.1007/s10856-008-3607-y

[12] Iwasaki Y Tojo Y Kurosaki T Nakabayashi N Reduced adhesion of blood cells to biodegradable polymers by introducing phosphorylcholine moieties J. Biomed. Mater. Res 2003 65A 164 169 10.1002/jbm.a.10459 12734808

[13] Chen NC Chen YW Wang LJ Luo XL Preparation, characterization and blood compatibility of polylactide-based phospholipid polymer J. Mater. Sci 2009 44 6317 6320

[14] Woo SI Kim BO Jun HS Chang HN Polymerization of aqueous lactic acid to prepare high molecular weight poly (lactic acid) by chain-extending with hexamethylene diisocyanate Polym. Bull 1995 35 415 421

[15] Nederberg F Bowden T Hilborn J Induced surface migration of biodegradable phosphorylcholine functional poly (trimethylene carbonate) Polym. Adv. Technol 2009 16 108 112

[16] Yang S Zhang SP Winnik FM Mwale F Gong YK Group reorientation and migration of amphiphilic polymer bearing phosphorylcholine functionalities on surface of cellular membrane mimicking coating J. Biomed. Mater. Res 2009 84A 837 841 10.1002/jbm.a.31418 17635030

